# Comparison of Eight Methods for the Extraction of *Bacillus atrophaeus* Spore DNA from Eleven Common Interferents and a Common Swab

**DOI:** 10.1371/journal.pone.0022668

**Published:** 2011-07-26

**Authors:** Helen L. Rose, Caroline A. Dewey, Morgan S. Ely, Sarah L. Willoughby, Tanya M. Parsons, Victoria Cox, Phillippa M. Spencer, Simon A. Weller

**Affiliations:** Detection Department, Defence Science and Technology Laboratory, Porton Down, Salisbury, United Kingdom; Instituto Butantan, Brazil

## Abstract

Eight DNA extraction products or methods (Applied Biosystems PrepFiler Forensic DNA Extraction Kit; Bio-Rad Instagene Only, Bio-Rad Instagene & Spin Column Purification; EpiCentre MasterPure DNA & RNA Kit; FujiFilm QuickGene Mini80; Idaho Technologies 1-2-3 Q-Flow Kit; MoBio UltraClean Microbial DNA Isolation Kit; Sigma Extract-N-Amp Plant and Seed Kit) were adapted to facilitate extraction of DNA under BSL3 containment conditions. DNA was extracted from 12 common interferents or sample types, spiked with spores of *Bacillus atropheaus*. Resulting extracts were tested by real-time PCR. No one method was the best, in terms of DNA extraction, across all sample types. Statistical analysis indicated that the PrepFiler method was the best method from six dry powders (baking, biological washing, milk, plain flour, filler and talcum) and one solid (Underarm deodorant), the UltraClean method was the best from four liquids (aftershave, cola, nutrient broth, vinegar), and the MasterPure method was the best from the swab sample type. The best overall method, in terms of DNA extraction, across all sample types evaluated was the UltraClean method.

## Introduction

The Polymerase Chain Reaction (PCR) is commonly used to detect pathogens from various sample types [1], [2]. Prior to performing PCR, DNA must be extracted efficiently from samples. An optimal extraction procedure will efficiently extract DNA from any micro-organism present in the sample whilst at the same time removing any protein, compound or chemical which may subsequently inhibit the PCR.

In order to be used confidently, DNA extraction techniques must process different sample types (i.e. dry powders, liquids, solids, swabs) of potentially unknown composition. For instance, talcum powder has been reported to be a common sample received by the US Bioterrorism Rapid Response and Advanced Technology (BRRAT) Laboratory [3]. As such samples are suspected of containing pathogens, initial processing of samples is often required to be conducted under Biological Safety Level 3 (BSL3) conditions or higher. BSL3 laboratory facilities are known to place ergonomic restrictions on operatives and general molecular biology practice [4], and therefore it is important that any DNA extraction method is as easy to use with a low as possible logistic and operative burden.

The aim of this study was to find a single method, suitable for use at BSL3, able to efficiently extract DNA from the spores of *Bacillus atrophaeus* spiked into various household interferents which represent a range of common environmental sample types (dry powders, liquids, solids, swabs). This will provide higher confidence that a selected extraction method can deal with sample types of unknown composition and thereby reduce the number of repeat extractions required due to failure of sub-optimal techniques. Eight commercial kits or products were adapted to address this aim.

## Materials and Methods

### Selection of sample types

Six powder samples types, four liquids, one solid sample type were selected for this study (Table 1) in addition to a common swab type. These products were chosen to cover a representative range of sample types whilst also including those thought to be challenging matrices from which to recover bacterial DNA, prior to PCR.

**Table 1:** Sample types used in this study.

Sample type	Product description	Listed ingredients (as and where stated by manufacturer)
Powder	Biological washing powder	Zeolite, oxygen based bleaching agent, anionic surfactant, non-ionic surfactant, polycarboxylate phosphonate, enzymes, optical brightener, perfume, butylphenyl methylpropional citronellol
Powder	Skimmed milk powder	Dried skimmed milk (99.5%), Vitamins A + D
Powder	Plain flour	*None listed*
Powder	Baking powder	Raising agent (disodium phosphate, sodium bicarbonate), rice flour
Powder	Talcum powder	Talc, parfum
Powder	Filler (spackling) powder	*None listed*
Liquid	Aftershave	Alcohol denat, aqua, parfum, PEG 40, hydrogenated caster oil, benzyl alcohol, benzyl benzoate, benzyl salicylate, citral, citronellol, coumarin, eugenol, geraniol, butylphenyl, methylpropional, limonene, linalool, hydroxisohexyl 3–cyclohexane carboxaldhye, alpha – isomethyl, ionone, evernia prunastr extract, evernia furfuracea extract
Liquid	Cola drink	Carbonated water, sugar, colour (caramel E150D), phosphoric acid, flavourings (including caffeine)
Liquid	Nutrient broth	Yeast extract, peptone, glucose, sodium chloride
Liquid	Malt vinegar	Barley malt vinegar, roast barley malt extract
Solid	Underarm deodorant	Cyclomethicone, aluminum zirconium tetrachlorohydrex GLY, PPG – 14 butyl ether, stearyl alcohol, hydrogenated caster oil, talc, PEG – 8 distearate, parfum, BHT, butylphenyl methylpropional, citronellol, coumarin, geraniol, hexyl cinnamal, limonene, linalool

### Spiking of sample types and quantities from which DNA was extracted

All sample types were spiked with the BI-CHEM™ MicroTrace™ (Novozymes Biologicals Inc, Salem, USA) preparation of dried spores of *Bacillus atropheaus* (termed Bg to relate to the previous name of this organism, *Bacillus globigii*). This product has a stated minimum spore concentration of 1×10^11^ colony forming units (cfu) /gram and was mixed with each sample type as described below:

#### Dry Powders

10% and 0.1% Bg/powder, weight/weight (w/w), samples were prepared for each powder type. These samples were stored in universal tubes at 4°C prior to DNA extraction and PCR. The same samples were used for all extracts prepared. As the 2001 anthrax attacks in the USA reportedly consisted of *B. anthracis* spore preparations containing at least 1×10^11^ spores/gram [5], DNA was also extracted directly from the MicroTrace™ product to simulate this sample type. Samples were stored at 4°C. Before extraction each universal tube was placed on a roll bar agitator for 10 minutes to ensure thorough mixing of the spore/powder mix. DNA was then extracted from 1 µL microbiological loops of each spiked powder.

#### Liquids

0.1% and 0.001% weight/volume (w/v) Bg/liquid samples were prepared in each liquid type. Aliquots (2 mL) were immediately stored at −20°C to ensure spores did not germinate to a vegetative cell state in each liquid sample type. At testing spiked liquid samples were thawed, vortexed, and DNA was immediately extracted from 100 µL aliquots.

#### Solid

Deodorant was grated to facilitate DNA extraction. The sticky consistency of this sample type plus the larger particle size did not allow an equal distribution of Bg spores when mixed with the MicroTrace™ product as described for powders. Therefore the capability of each method in removing PCR inhibitors from this sample type was determined by extracting DNA from 100 µL of a 0.1% w/v Bg spore/sterile distilled water preparation in the presence of a 1 µL loop of the grated deodorant. DNA extraction then proceeded using extraction protocols for liquids (see below), with the initial lysis reagents being added directly to this tube.

#### Swab

To re-create a typical swab sample, a BSL1 containment cabinet was dry swabbed with a cotton swab (150C Cotton swab with wood stem, COPAN Italia S.p.A., Brescia 25125, Italy). Each swab was re-hydrated in a 150 µl aliquot of a 0.001% (w/v) Bg spore/1× Phosphate Buffer Saline (PBS) suspension. The cotton end of the swab was then cut off and placed in a Swab Extraction Tube System (Roche Diagnostics GmbH, Mannheim, Germany). This tube was centrifuged (10 000 rpm; 3 min) and the resulting eluent removed. DNA was extracted from each eluent using extraction protocols for liquids (100 µL sample volume).

### DNA extraction methods

Eight commercial kits were evaluated in this study, comprising several different methodologies. Manufacturers protocols were adapted to increase DNA extraction efficiency and also facilitate ease of use in BSL3 cabinets. A limit of two medium sized pieces of equipment (i.e. heat-blocks, microfuges) was imposed on each method. DNA was initially extracted from 15 replicates of each sample type/Bg concentration combination by some of the methods. However, a statistical review of initial results indicated that, for the remaining methods, this could be reduced to 9 replicates without a loss in the power of the analysis.

#### Instagene Only

One µL loop of powder sample was added to 1 mL Instagene™ Matrix (Bio-Rad, Hercules, CA, USA) or 100 µL liquid sample was added to 900 L of Instagene. Instagene/sample suspension was heated (95°C; 15 min) and centrifuged (10 000 rpm; 3 min). Supernatant was retained for PCR.

#### Spin Column Purification of Instagene supernatant

Reagents (S3, S4, & S5) and spin columns from UltraClean™ Soil DNA Kits (MO BIO Laboratories Inc., Carlsbad, USA) were used. Four hundred µL of Instagene supernatant (from Instagene Only protocol) was added to 800 L of solution S3. Two ×600 L aliquots of supernatant/S3 solution were added to the spin column and centrifuged 10 000 rpm; 30 sec. Each flow through was discarded. Three hundred µL of solution S4 was added to the spin column, centrifuged (10 000 rpm; 1 min), and flow through discarded. Two-hundred and fifty µL of solution S5 was added to the spin column, centrifuged (10 000 rpm; 1 min) and the flow through retained for PCR.

#### Sigma Extract-N-Amp Plant and Seed Kit

Reagents from Extract-N-Amp™ Plant and Seed Kit (Sigma-Aldrich, St. Louis, MO, USA) were used with a method adapted from a previous study [6], For powders, a 1 µL loop of sample was added to 45 µL of Extraction solution and 5 µL seed preparation solution and incubated (55°C, 10 min, then 95°C, 10 min). Fifty µL of neutralisation solution was then added and the entire suspension diluted 1∶20 (in sterile distilled water) and retained for PCR. For liquids, 100 µL of sample was added to 90 µL Extraction solution and 10 µL seed preparation solution. After incubation (as above), 100 µL of neutralisation solution was added prior to diluting 1∶20 for PCR.

#### UltraClean™ Microbial DNA Isolation Kit

Reagents (MD2, MD3, MD4, & MD5) and spin columns from UltraClean™ Microbial DNA Isolation Kits (MO BIO Laboratories Inc., Carlsbad, USA) were used. One µL loopful of powder, or 100 µL of liquid sample, was added to 300 µL of Microbead Solution (without beads) and 50 µL of MD1 solution. Suspensions were heated (95°C, 15 min) and centrifuged (10 000 rpm, 2 min). Three hundred µL of supernatant was added to 100 µL of MD2 solution, tubes were inverted several times, incubated (20°C, 5 min), and centrifuged (10 000 rpm, 2 min). Three hundred µL of the supernatant was then added to 900 µL of MD3 solution****. Two ×600 µL aliquots of supernatant/MD3 solution were sequentially added to the spin column and centrifuged (10 000 rpm; 30 sec). Each flow through was discarded. Three hundred µL of solution MD4 was then added to the spin column, centrifuged as above and flow through discarded. Two-hundred and fifty µL of solution MD5 was then added to the spin column, centrifuged (10 000 rpm; 1 min) and the flow through retained for PCR.

#### Idaho Technologies 1-2-3 Q-Flow

Reagents (AL, AW1, AW2 and AE) and spin columns from 1-2-3 Q-Flow kits (Idaho Technologies Inc, Salt Lake City, UT, USA), based on Qiagen technology, were used. One µL loopful of powder was added to 300 µL 1 ×PBS and 300 µL AL buffer, or 100 µL loopful of liquid sample to 200 µL 1 ×PBS and 300 µL AL buffer. Mixtures were incubated (95°C, 15 min). Three hundred µL of ethanol was added and tubes inverted several times. Two ×450 µL aliquots of the resulting lysate were added sequentially to a spin column and centrifuged (10 000 rpm; 30 sec). Each flow-through was discarded. Five hundred µL of AW1 buffer was added to the spin column, centrifuged as above and flow through discarded. Five hundred µL of AW2 buffer was added to the spin column, centrifuged (10 000 rpm; 1 min) and the flow-through discarded. Two hundred and fifty µL of AE buffer was then added to spin column, centrifuged (10 000 rpm; 1 min) and the flow-through retained for PCR.

#### FujiFilm QuickGene Mini80

Reagents (MDT, LDT, WB, & EB) and cartridges from FujiFilm QuickGene DNA Tissue Kit S and QuickGene Mini80™ Nucleic Acid Isolation Device (FujiFilm Corporation, Tokyo, Japan) were used. One µL loopful of powder was added to 750 µL MDT buffer, or 100 µL of liquid sample added to 650 µL of MDT buffer. Mixtures were incubated (95°C, 10 min) and the tube inverted several times. Four hundred µL of supernatant added to 360 µL of LDT buffer and 480 µL of ethanol. Six hundred and twenty µL of this lysate was added to a QuickGene cartridge and the system pressurised to pass lysate through the cartridge membrane. The remaining lysate was added and the system re-pressurised. The cartridge was then washed sequentially with 3×750 µL aliquots of WB buffer. Two hundred and fifty µL of EB buffer was then added to the cartridge. The Mini80 manifold was placed above a collection tube, the system was re-pressurised, and the flow through retained for PCR.

#### Revised FujiFilm QuickGene Mini80 Method

At the end of the experimental work a potential improvement to this method was identified – adding a 1 µL loopful of powder to 500 µL MDT buffer, or 100 µL of liquid sample to 400 µL of MDT buffer. These mixtures were then processed as above.

#### Applied Biosystems PrepFiler™ Kit

Reagents from PrepFiler™ Forensic DNA extraction kit and a 6 Tube Magnetic Stand (Applied Biosystems, Foster City, CA, USA) were used. One µL loopful of powder was added to 300 µL Lysis Buffer and 3 µL DL-Dithiothreitol (DTT), or 100 µL of liquid sample was added to 300 µL Lysis Buffer and 4 µL DTT. Mixtures were incubated (80°C, 15 min). Fifteen µL of re-suspended PrepFiler Magnetic Particles and 180 µL isopropanol were added to tubes. Tubes were agitated for 10 minutes and then inverted several times. Tubes were then placed on the magnetic stand. Buffer was removed from each tube once the pellet had formed (without disturbing the pellet). Three ×300 µL aliquots of Wash Buffer were then added sequentially. At each application the pellet was re-suspended, the tube placed back on Magnetic Stand, the pellet allowed to reform, and the Wash Buffer removed and discarded. After the final step it was ensured that all Wash Buffer was removed from tube by a pulse centrifugation step and re-application of the tube to the magnetic stand, which allowed any remaining Wash Buffer to be removed and discarded. Two hundred and fifty µL of Elution Buffer was then added, and the pellet re-suspended and incubated (70°C; 5 min). The tube was then placed back on stand, the pellet allowed to re-form and buffer was retained for PCR.

#### Epicentre MasterPureKit

Reagents from the MasterPure™ Complete DNA and RNA Purification Kit (Epicentre Biotechnologies, Madison, WI, USA) were used. One µL loopful of powder was added to 300 µL Tissue & Cell Lysis solution, or 100 µL of liquid sample was added to 200 µL Tissue & Cell Lysis solution. Mixtures were incubated (95°C; 15 min). One hundred and seventy five µL MPC solution was added and cell debris was pelleted by centrifugation (13 000 rpm; 10 min). The supernatant was removed to clean tube, 500 µL of isopropanol added, and DNA pelleted by centrifugation (13 000 rpm; 10 min). The supernatant was removed and the pellet washed sequentially with 2×500 µL aliquots of ethanol. All ethanol was removed from the tube and the pellet re-suspended in 250 µL of 1× Tris-EDTA (TE) buffer which was then retained for PCR.

### Real-time PCR analysis

The real-time PCR (Bg sp) assay [7] was used to compare the ability of each method to extract *B. atrophaeus* DNA from the different spiked samples. PCR primers and probe for this assay were purchased (ATDBio Ltd., Southampton, UK). The Bg sp probe was covalently labelled at the 5′ end with the reporter dye FAM and at the 3′ end with the quencher dye BHQ-1^TM^. Real-time PCRs (24 µL volume) comprised 12 µL DNA extract, Bg sp Forward primer (900 nM), Bg sp Reverse primer (300 nM), Bg sp Probe (200 nM) and PCR mastermix containing 0.04 units/µl JumpStart™ Taq DNA polymerase (Sigma-Aldrich Company Ltd., Gilllingham, UK), 2 µM dNTPs, 8% w/v glycerol, 4 mM MgCl_2,_ 50 mM Tris-HCl_,,_ 1 µg/µL BSA, 0.5 µM EGTA. PCR cycling conditions comprised 3 min at 95°C, 30 s at 60°C, followed by 50 two-step cycles of 15 s at 95°C and 30 s at 60°C.

### Statistical Analysis

Using a scoring and ranking method, commonly used in statistical practice, the raw data (each C_t_ value returned by each PCR from each individual DNA extract, not shown), was analysed. To incorporate the negative/positive result ratio into this analysis, negative results were assigned a C_t_ value of 50 (the PCR cycle cut-off). The sum of these scores was taken, giving one score per method. These were then ranked (with lowest values highest to indicate either an increase in DNA yield or absence of PCR inhibition between methods). It should be noted that for methods which generated identical scores, the variance of the score was taken into account. The method with the least variance was then ranked highest.

## Results

### Powders

Results from powders are summarised in Table 2. All the methods produced extracts that resulted in positive PCR results when taken from the 10% Bg/powder sample types. The 0.1% Bg/powder samples helped indicate methods which were less able to remove PCR inhibitors from the resulting extracts or resulted in reduced yields of DNA. Instagene only DNA extracts resulted in only 3 (from 15) PCR positives from baking powder; Extract-N-Amp extracts from filler powder resulted in 5/15 positives, Masterpure extracts from both filler and talc powders resulted in 4/9 PCR positives, and the QuickGene method produced 6/9 PCR positives from both the flour and filler samples. The revised QuickGene method produced 9/9 positives for both flour and filler samples types (Table 3). A t-test indicated that for plain flour the revised QuickGene method produced significantly better results (in terms of lower Ct values) than the original QuickGene method. For filler powder results (in terms of Ct values) were not significantly different, between the original and revised methods, although the confidence intervals were tighter for the revised method (analyses not shown). The PrepFiler method was ranked the best method from powders by statistical analysis.

**Table 2:** Results from Bg sp PCR when tested against DNA extracts produced from powder sample types spiked with two concentrations of *B. atropheaus.*

Powder sample	DNA extraction method															
	Instagene™ Only		Instagene™ & Spin Column		Extract-N-Amp™ Plant & Seed		UltraClean™ Microbial DNA		1-2-3 Q-Flow™		QuickGene Mini80™		Masterpure™ DNA & RNA		PrepFiler™ Forensic DNA	
	+ ve's^b^	Mean C_T_c	+ ve's	Mean C_T_	+ ve's	Mean C_T_	+ ve's	Mean C_T_	+ ve's	Mean C_T_	+ ve's	Mean C_T_	+ ve's	Mean C_T_	+ ve's	Mean C_T_
Baking 10% Bg^a^	15/15	37.8 (10.7)	15/15	24.3 (0.6)	15/15	29.2 (2.9)	9/9	26.5 (0.7)	9/9	22.2 (0.4)	9/9	28.7 (1.0)	9/9	25.9 (0.1)	9/9	19.7 (1.4)
Baking 0.1% Bg	3/15	41.9 (8.8)	15/15	30.8 (1.0)	13/15	37.5 (4.2)	9/9	34.1 (1.1)	9/9	31.9 (3.2)	9/9	37.1 (2.1)	8/9	32.9 (1.2)	9/9	29.8 (10.4)
Bio-wash 10% Bg	15/15	19.0 (0.2)	15/15	17.5 (0.9)	15/15	22.4 (0.2)	9/9	19.8 (0.1)	9/9	20.0 (1.3)	9/9	22.0 (0.6)	9/9	22.4 (8.1)	9/9	19.2 (0.4)
Bio-wash 0.1% Bg	15/15	27.2 (1.5)	15/15	25.1 (2.3)	15/15	30.5 (3.6)	9/9	30.9 (10.5)	9/9	28.9 (8.1)	8/9	31.5 (5.0)	9/9	29.5 (9.7)	9/9	30.7 (24.1)
Filler 10% Bg	15/15	25.5 (0.6)	15/15	26.0 (0.7)	15/15	31.3 (2.1)	9/9	28.4 (2.6)	9/9	24.7 (2.1)	9/9	29.0 (1.5)	9/9	31.7 (2.6)	9/9	20.5 (0.4)
Filler 0.1% Bg	14/15	35.1 (3.0)	15/15	34.9 (3.7)	5/15	35.7 (38.3)	7/9	35.9 (4.6)	7/9	34.0 (7.1)	6/9	35.2 (3.4)	4/9	39.2 (2.5)	9/9	32.0 (3.7)
Flour 10% Bg	15/15	26.0 (0.4)	15/15	23.8 (0.4)	15/15	27.5 (9.3)	9/9	22.6 (0.7)	9/9	23.5 (1.0)	9/9	25.5 (0.8)	9/9	21.9 (1.0)	9/9	24.1 (1.0)
Flour 0.1% Bg	15/15	33.0 (2.9)	15/15	29.2 (3.4)	15/15	31.3 (4.8)	9/9	30.1 (4.5)	9/9	32.0 (6.7)	6/9	34.0 (1.1)	9/9	30.9 (16.5)	9/9	30.9 (1.7)
Milk 10% Bg	15/15	26.0 (0.44)	15/15	24.3 (0.4)	15/15	25.6 (1.3)	9/9	22.2 (0.6)	9/9	21.2 (1.2)	9/9	24.4 (1.0)	9/9	20.6 (0.7)	9/9	20.5 (0.1)
Milk 0.1% Bg	15/15	30.6 (0.9)	14/15	32.0 (0.6)	15/15	34.0 (1.9)	9/9	29.2 (1.5)	9/9	27.8 (1.0)	9/9	30.9 (3.4)	9/9	27.6 (2.9)	9/9	28.0 (4.4)
Talcum 10% Bg	15/15	26.6 (2.2)	15/15	27.0 (0.8)	15/15	27.1 (4.3)	9/9	28.0 (1.6)	9/9	24.1 (2.9)	9/9	27.4 (2.9)	9/9	32.4 (2.7)	9/9	26.2 (3.0)
Talcum 0.1% Bg	15/15	32.7 (4.8)	15/15	33.3 (1.2)	12/15	34.7 (6.6)	9/9	34.3 (14.8)	9/9	33.5 (4.5)	9/9	34.7 (10.8)	4/9	37.5 (12.1)	8/9	36.1 (0.9)
BI-CHEM™ MicroTrace™	15/15	18.3 (0.1)	15/15	18.3 (0.2)	15/15	23.8 (0.3)	9/9	24.7 (12.3)	9/9	20.7 (1.0)	9/9	22.2 (0.1)	9/9	21.2 (0.1)	9/9	19.7 (0.2)

a weight/weight powder/Bg spores.

b No. of PCR positives from *n* replicates.

c C_T_ value: PCR cycle number at which fluorescence first detected in a 50 cycle PCR. Mean of positive results only. Variance of mean in parenthesis.

**Table 3:** Results from Bg sp PCR when tested against DNA extracts produced from 0.1% Bg w/w in flour and filler samples types (9 replicates) using the original and revised Quickgene method.

Sample	Extraction method	Rep 1 C_T_ b	Rep 2 C_T_	Rep 3 C_T_	Rep 4 C_T_	Rep 5 C_T_	Rep 6 C_T_	Rep 7 C_T_	Rep 8 C_T_	Rep 9 C_T_	Mean C_T_	Var. of Mean C_T_
0.1% Bg/Flour^a^	QuickGene	35.15	34.2	33.84	34.81	-	32.27	-	33.49	-	34.0	1.1
0.1% Bg/Flour	Revised QuickGene	30.25	33.82	31.64	32.84	30.76	30.94	30.95	31.4	34.8	31.9	2.4
0.1% Bg/Filler	QuickGene	-	-	35.7	37.77	32.29	29.78	37.75	37.75	-	35.2	3.4
0.1% Bg/Filler	Revised QuickGene	33.45	36.44	33.68	38.2	34.77	37.11	32.38	37.6	33.58	35.2	4.5

a w/w Bg/powder.

b C_T_ value: PCR cycle number at which fluorescence first detected in a 50 cycle PCR. (-)  =  negative result.

### Liquids

Results from liquids are summarised in Table 4. The UltraClean, 1-2-3 Q-Flow, and QuickGene and Instagene & Spin Column methods resulted in PCR positives from all (or nearly all) replicates tested. Of these methods the UltraClean method was ranked best by statistical analysis.

**Table 4:** Results from Bg sp PCR when tested against DNA extracts produced from liquid sample types spiked with two concentrations of *B. atropheaus* spores.

Liquid Sample	DNA Extraction Method															
	Instagene™ Only		Instagene™ & Spin Column		Extract-N-Amp™ Plant & Seed		UltraClean™ Microbial DNA		1-2-3 Q-Flow™		QuickGene Mini80™		Masterpure™ DNA & RNA		PrepFiler™ Forensic DNA	
	+ ve's^b^	Mean C_T_ c	+ ve's	Mean C_T_	+ ve's	Mean C_T_	+ ve's	Mean C_T_	+ ve's	Mean C_T_	+ ve's	Mean C_T_	+ ve's	Mean C_T_	+ ve's	Mean C_T_
AfterShave 0.1% Bg^a^	15/15	28.9 (6.2)	15/15	23.9 (0.2)	15/15	28.4 (0.2)	9/9	26.6 (2.4)	9/9	24.5 (0.4)	9/9	29.8 (2.2)	9/9	27.8 (10.66)	8/9	27.1 (40.4)
AfterShave 0.001% Bg	15/15	34.5 (1.7)	15/15	31.3 (0.1)	15/15	35.2 (0.2)	9/9	33.3 (1.5)	9/9	31.9 (0.6)	9/9	32.5 (0.6)	7/9	31.3 (0.4)	4/9	26.6 (13.6)
Cola Drink 0.1% Bg	0/15	−	15/15	24.7 (0.3)	14/15	28.3 (0.3)	9/9	28.5 (0.1)	9/9	27.3 (1.0)	9/9	27.3 (0.4)	9/9	32.1 (4.1)	9/9	29.1 (0.8)
Cola Drink 0.001% Bg	0/15	−	15/15	32.0 (0.2)	15/15	35.3 (0.7)	9/9	34.3 (0.5)	9/9	33.0 (11.0)	9/9	33.9 (2.0)	4/9	47.0 (8.5)	9/9	37.1 (6.3)
N-Broth 0.1% Bg	15/15	25.1 (0.04)	14/15	24.2 (0.1)	15/15	27.8 (0.1)	9/9	25.8 (0.1)	9/9	25.1 (0.1)	9/9	26.4 (0.6)	9/9	24.1 (0.02)	9/9	26.9 (1.1)
N-Broth 0.001% Bg	15/15	31.0 (0.2)	15/15	32.3 (0.1)	15/15	34.8 (1.4)	8/8	31.9 (0.1)	9/9	31.8 (0.2)	9/9	32.3 (6.2)	9/9	31.3 (0.1)	9/9	35.1 (0.3)
Vinegar 0.1% Bg	13/15	41.3 (14.2)	14/15	36.6 (1.7)	7/15	40.5 (9.4)	9/9	26.2 (0.1)	9/9	32.0 (0.2)	9/9	27.4 (0.8)	8/9	32.6 (7.3)	9/9	27.2 (1.2)
Vinegar 0.001% Bg	1/15	41.9	15/15	36.7 (0.8)	10/15	39.1 (3.9)	9/9	33.4 (0.2)	9/9	38.3 (10.6)	9/9	33.5 (0.8)	2/9	36.4 (0.1)	9/9	35.1 (0.3)

a weight/volume Bg spores/liquid.

b No. of PCR positives from *n* replicates.

c C_T_ value: PCR cycle number at which fluorescence first detected in a 50 cycle PCR. Mean of positive results only. Variance of mean in parenthesis.

The PrepFiler method produced PCR positives from all sample types except the aftershave 0.001% Bg sample type where 4/9 PCR positives were observed. The other methods produced repeated negatives in at least two of the other sample types, notably Instagene only extracts from cola where no PCR positives from 30 extracts were returned.

### Solid

Results from Bg DNA extracted in the presence of the solid sample type are summarised in Table 5. This sample type produced the most inhibition when compared with the other sample types. Only the PrepFiler and UltraClean methods returned PCR positives (with low and consistent C_t_ values), from all extracts generated. The PrepFiler method was ranked best for the solid type.

**Table 5:** Results from Bg sp PCR when tested against DNA extracts produced from a 100 µL (0.001% w/v) Bg aliquot (with an added 1 µL microbiological loopful of grated deodorant) and 100 µL (0.001% w/v) Bg aliquots removed from a cotton swab.

Sample	DNA Extraction Method															
	Instagene™ Only		Instagene™ & Spin Column		Extract-N-Amp™ Plant & Seed		UltraClean™ Microbial DNA		1-2-3 Q-Flow™		QuickGene Mini80™		Masterpure™ DNA & RNA		PrepFiler™ Forensic DNA	
	+ ve's^1^	Mean C_T_ b	+ ve's	Mean C_T_	+ ve's	Mean C_T_	+ ve's	Mean C_T_	+ ve's	Mean C_T_	+ ve's	Mean C_T_	+ ve's	Mean C_T_	+ ve's	Mean C_T_
Deodorant	0/15	−	0/15	−	11/15	38.7 (4.4)	9/9	24.2 (7.1)	6/9	35.9 (24.5)	3/9	37.4 (0.9)	1/9	39.3	9/9	24.0 (1.5)
Swab	15/15	36.9 (0.3)	15/15	36.2 (0.4)	12/15	39.8 (5.0)	9/9	36.6 (1.9)	8/9	36.2 (0.9)	8/9	37.6 (1.4)	9/9	35.5 (2.0)	5/9	40.3 (16.3)

a No. of PCR positives from *n* replicates.

b C_T_ value: PCR cycle number at which fluorescence first detected in a 50 cycle PCR. Mean of positive results only. Variance of mean in parenthesis.

### Swabs

Results from Bg DNA extracted from a 0.001% Bg spore suspension removed from cotton swabs are summarised in Table 5. With the exception of the Extract-N-Amp and PrepFiler methods all methods produced PCR positives from all, or all but one, DNA extracts generated. The MasterPure method was ranked best for the swab sample type by statistical analysis.

## Discussion

This study is an attempt to find a single method suitable for extracting DNA, under BSL3 containment conditions, from a range of different samples types prior to the application of PCRs which are targeted to various pathogens. To the best of our knowledge this is the largest study in terms of numbers of methods and interferents yet published. Each method was adapted from protocols suggested by the manufacturer rather than in other reports where the authors have generally followed manufacturers instructions [2], [3], [8]. In our hands no one method was shown to be the best for each of the sample types (dry powders, liquids, solids, swabs) tested.

Various approaches to adapting each kit were undertaken in the initial method development stage of the project. It was found that the addition of a heat step at the beginning of the process (>80°C, ≥10 min) gave better results than other approaches to spore lysis such as bead beating or chemical lysis (data not shown). We presume this is because a heat step is the best way to ensure spore degradation, releasing more intracellular DNA into the extraction mix. Thus all methods described in this report use such a heat step even if not suggested by the manufacturer. Other components of kits were also discarded where possible, notably the use of Proteinase K which was not compatible (or necessary) with the addition of a heat step.

Sample types were chosen to give a broad range of challenging matrices and thus increase overall confidence any down selected method. All methods generally performed well with powder and swab samples. The PrepFiler method gave the lowest C_t_ values from the powder and solid sample types, the UltraClean method gave the best results from liquids and the MasterPure method gave the best result from swabs, as observed in a previous study [3]. Sample types which gave less optimal results from some of the lesser performing methods included cola drink, vinegar and underarm deodorant. The deodorant sample type was the most challenging sample type in this study with only the PrepFiler and UltraClean methods generating consistent and reproducible results. Statistical analysis indicated that the best overall method for DNA Extraction was the UltraClean method (Table 6).

**Table 6:** Ranking of each method, as determined by statistical analysis, by individual sample type and combined sample types.

Rank	Powders	Liquids	Solid	Swab	Combined
1	PrepFiler Forensic DNA	Ultraclean Microbial DNA	PrepFiler Forensic DNA	Masterpure DNA & RNA	Ultraclean Microbial DNA
2	1-2-3 Q-Flow	Quickgene Mini80	Ultraclean Microbial DNA	Instagene & Spin Column	1-2-3 Q-Flow
3	Instagene & Spin Column	1-2-3 Q-Flow	1-2-3 Q-Flow	Ultraclean Microbial DNA	PrepFiler Forensic DNA
4	Ultraclean Microbial DNA	Instagene & Spin Column	Extract-N-Amp Plant & Seed	Instagene Only	Instagene & Spin Column
5	Instagene Only	PrepFiler Forensic DNA	Quickgene Mini80	1-2-3 Q-Flow	Masterpure DNA & RNA
6	Masterpure DNA & RNA	Masterpur DNA & RNA	Masterpure DNA & RNA	Quickgene Mini80	Quickgene Mini80
7	Quickgene Mini80	Extract-N-Amp Plant & Seed	Instagene Only	Extract-N-Amp Plant & Seed	Instagene Only
8	Extract-N-Amp Plant & Seed	Instagene Only	Instagene & Spin Column	PrepFiler Forensic DNA	Extract-N-Amp Plant & Seed

In terms of ease of use, staff determined the Instagene Only and QuickGene Mini80 methods as being the easiest to use. Neither of these methods required multiple manipulations, such as repeated removal of spin columns from tubes. It was determined that the MasterPure method was the most difficult to use. This method involves the production of a tiny DNA pellet in a 1.5 mL microtube and it was considered that this would not be routinely practicable in a BSL3 cabinet. Of the spin column methods, staff found the architecture of the 1-2-3 Q-Flow columns to be easier to use than those from the UltraClean kit.

Unlike other studies [3], [8], [9], we have not defined the limits of detection of each method/sample type combination in terms of number of cells per gram or millilitre of sample. This is primarily because we used a simulant rather than an actual pathogen in our spiked samples types and also because many factors influence infectious doses from the sample types in question [10]. However, the commercial preparation of Bg stimulant used in our study mimicked, in terms of composition and number of cells per gram, the *B. anthracis* spore preparation reportedly used in the 2001 anthrax attacks in the USA [5]. In powders, we have shown that the best methods can robustly detect this simulant in a 1∶1000 w/w spores/powder ratio. Observing the assay process as a whole, taking into account the mean Ct values generated and assuming that an increase of 3 Ct values is equivalent to a 10-fold decrease in agent concentration, it seems possible that the best methods could detect even lower concentrations of agent within the powder types. All methods robustly extracted DNA from the neat spore preparation (Table 2), indicating that increased amounts of DNA did not inhibit the resulting PCR, as has been seen from some sample types previously [11].

This study should be used in conjunction with other studies [2], [3], [8], [12] to identify the most appropriate DNA extraction method for a particular set of local requirements/facilities. The protocols described in this paper are one representation of what can be achieved with the components of each kit. Other groups may be able to find more appropriate methods which utilise these kits to fit their own local requirements and also improve upon the results presented. Indeed, with the experience gained during this study we readily identified an improvement to the QuickGene Mini80 method by decreasing the amount of MDT buffer and ensuring more of the DNA from the sample is processed. In addition, variants of the same kits may also produce better results. DNA was only extracted from bacterial spores in this study. Potential pathogens which take the form of vegetative cells, virions or fungal hyphae are also known to exist. Therefore, for a full understanding of performance DNA extraction method should also be tested against these types of organisms.

In this study over two thousand DNA extracts were produced and PCRs conducted to evaluate eight methods against 12 common interferents. These interferents were selected to give as broad a challenge (in terms of different PCR inhibitors), to each method and provide as much confidence as possible that any down-selected method could deal with the unknown sample. Indeed, it is unlikely that a sample such as vinegar would ever be tested. However, a user would have more confidence, when faced with an unknown sample, in using a method which had previously been shown to be able to deal with an extreme sample type (i.e. with very low pH) such as vinegar.

It is also true that more than 12 interferents exist and therefore it is impossible to validate methods against every unknown sample type. It is advisable that DNA extraction control methods are also developed and applied to samples to obtain a higher confidence in PCR results. A previous study reported the development of such a control by adding *Bacillus atrophaeus* (Bg) spores to vaginal and anal diagnostic sample types and testing the resulting extract by a Bg specific PCR [13]. Combining a DNA extraction control (with appropriate non-target organism) with the best possible DNA extraction method would provide a system delivering extra confidence to PCR results, especially in reducing the possibility of false negative results.
